# Detection of testosterone administration based on the carbon isotope ratio profiling of endogenous steroids: international reference populations of professional soccer players

**DOI:** 10.1136/bjsm.2009.058669

**Published:** 2009-06-22

**Authors:** E Strahm, C Emery, M Saugy, J Dvorak, C Saudan

**Affiliations:** 1 Swiss Laboratory for Doping Analyses, University Center of Legal Medicine, West Switzerland, Epalinges, Switzerland; 2 FIFA Medical Assessment and Research Centre, Zürich, Switzerland

## Abstract

**Background and objectives:**

The determination of the carbon isotope ratio in androgen metabolites has been previously shown to be a reliable, direct method to detect testosterone misuse in the context of antidoping testing. Here, the variability in the ^13^C/^12^C ratios in urinary steroids in a widely heterogeneous cohort of professional soccer players residing in different countries (Argentina, Italy, Japan, South Africa, Switzerland and Uganda) is examined.

**Methods:**

Carbon isotope ratios of selected androgens in urine specimens were determined using gas chromatography/combustion/isotope ratio mass spectrometry (GC-C-IRMS).

**Results:**

Urinary steroids in Italian and Swiss populations were found to be enriched in ^13^C relative to other groups, reflecting higher consumption of C3 plants in these two countries. Importantly, detection criteria based on the difference in the carbon isotope ratio of androsterone and pregnanediol for each population were found to be well below the established threshold value for positive cases.

**Conclusions:**

The results obtained with the tested diet groups highlight the importance of adapting the criteria if one wishes to increase the sensitivity of exogenous testosterone detection. In addition, confirmatory tests might be rendered more efficient by combining isotope ratio mass spectrometry with refined interpretation criteria for positivity and subject-based profiling of steroids.

Anabolic androgenic steroids (AAS), particularly testosterone, are commonly used by athletes to enhance sports performance. The extent of misuse of AAS by competing athletes is clearly shown by the statistics of the adverse analytical findings reported by accredited laboratories in the last few years. Moreover, the same hormones that are included in the list of prohibited substances published by the World Anti-Doping Agency (WADA) are also misused by young people and non-competing amateurs.^1^ Chronic administration of AAS has been shown to produce endocrine, somatic and neuropsychiatric side effects. Psychological side effects are of particular concern because they induce violent behaviours with potentially serious consequences for the society at large.^1^

Testosterone doping cannot be detected by simply measuring the levels of endogenous hormones such as androgens in biological fluids. The variability in human metabolism of this compound is simply too large.^2^ In the antidoping field, evidence of testosterone administration relies on a confirmatory procedure that uses isotope ratio mass spectrometry (IRMS).^3^ A technical document established by WADA in 2004, stipulates that a urine sample must be analysed by IRMS to determine the carbon isotope ratios of androgens if the peak area ratio of testosterone/epitestosterone equivalent to the glucuronide is equal to or greater than 4.0 or if altered steroid profiles are determined.^4^ Endogenous testosterone is produced in the human body via cholesterol metabolism.^5^ ^6^ Detection of testosterone doping relies on the general observation that endogenous testosterone has a different ^13^C content compared to hemisynthetic testosterone used in pharmaceutical preparations. Such detection is possible because the carbon atoms in steroid molecules originate primarily in atmospheric CO_2_, which is fixed through photosynthesis. The most important photosynthetic pathways used by plants are the so-called C3 and C4 pathways.^7^ ^8^ Significant isotope fractionation occurs during photosynthetic carbon fixation, depending on the mode of CO_2_ fixation. Thus, the key enzymes that fix CO_2_ in C3 plants discriminate more strongly against ^13^CO_2_ than their analogues in C4 plants. As a result, the two types of plants differ by about 14‰ in the isotopic composition of their tissues. The natural abundance carbon isotopic ratio is expressed as a δ value relative to an international standard (Vienna Pee Dee Belemnite, VPDB; equation 1):


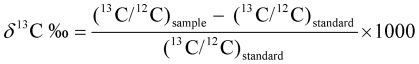


The practical outcome is the fact that the distribution of carbon isotopes in an animal reflects the relative abundance of food in the diet that originates directly or indirectly from C4 and C3 plants (either plants or animals that are lower in the food chain). Controlled diet studies have shown that the isotopic composition of the whole body of an animal is enriched by about 1‰ as a function of the isotopic composition of its diet.^9^

Although the diet composition of an athlete has a predominant influence on the carbon isotope ratio of the steroids excreted in urine, there have been no published comparisons of this diagnostic parameter in any cohort of elite athletes in a specific sports category. In this study, the range of the carbon isotope ratio of the steroids relevant to antidoping analysis was investigated in urine specimens obtained from top-level soccer player populations residing in six countries, namely Argentina, Italy, Japan, Republic of South Africa, Switzerland and Uganda. The determination of threshold values specific for a given diet and athlete metabolism is expected to significantly improve the detection of testosterone misuse by means of stable isotope methodology.

## Methods

### Population and sample collection

Urine samples were collected from 171 male soccer players aged from 18 to 36 (mean (SD) age 24.5 (3.8) years old). These elite athletes were competing in top-level teams in championships taking place in 6 different countries: Argentina (ARG, n = 31), Italy (ITA, n = 19), Japan (JAP, n = 32), The Republic of South Africa (SAF, n = 30), Switzerland (SWI, n = 31) and Uganda (UGA, n = 28). The study was initiated after validation of the protocol by the ethical committee of the University of Lausanne, Switzerland. A medical officer informed the participants in each soccer team about the goal of the project and the period of sampling. The subjects were not asked for a detailed description of their eating habits, but they were asked to sign a consent form which stipulated that they did not take any medicine that could influence their steroid profile for at least 4 weeks prior to urine collection. The urine samples were collected by the medical officer using BEREG-KITs (Berlinger AG, Ganterschwil, Switzerland). The anonymised samples were sent to the laboratory and the temperature was maintained at 4°C. Upon arrival, the samples were distributed in 20-ml glass containers and frozen at −20°C prior to extraction. The information made available to the laboratory included age, sex, country of residence, as well as the date and time of urine collection.

### Analytical methods

To reasonably ensure that no athlete used a doping agent prior to urine collection, all specimens were initially subjected to the analytical assays carried out in our WADA-accredited laboratory for the screening of substances prohibited in-competition.^10^

For the purpose of our study, a gas chromatography/combustion/isotope ratio mass spectrometry (GC-C-IRMS) method was used to determine the carbon isotope ratio of androsterone (A), etiocholanolone (Etio), 16(5α)-androstenol (16EN) and 5β-pregnanediol (PD).^11^ This assay, based on three solid phase extraction (SPE) cleanup steps, required an initial volume of urine of 10 ml. The urine samples used for the entire study were processed in different batches (n = 7) and included negative quality controls (QC). It is noteworthy that no drift was observed in the carbon isotope ratio of the analytes in the QC during the period of analysis (5 months) and the standard deviations reached 0.39‰, 0.34‰, 0.34‰ and 0.29‰ for Etio, A, 16EN and PD, respectively.

### Data analysis

All statistical analyses were performed using S-PLUS V. 7.0 (TIBCO Software Inc, Palo Alto, California, USA) for Windows. For distribution testing, the Kolmogorov–Smirnov test of normality was used. Testing of statistical differences among two normally distributed groups relied on the two-sample t test. A one-way analysis of variance (ANOVA) was applied to compare more than two groups. The level of significance was established at p<0.05 for all statistics.

## Results and discussion

**Figure 1:**
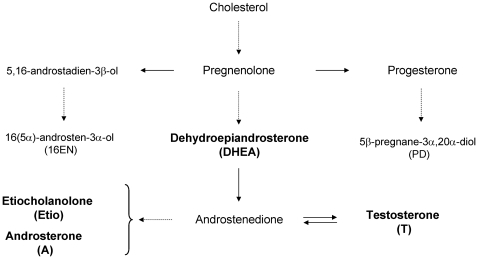
Simplified metabolic pathway of the steroids investigated in that study.

**Figure 2:**
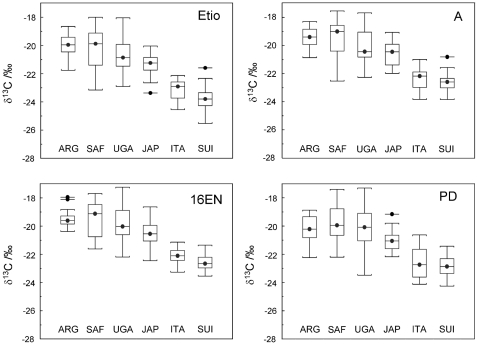
Distribution of d13C values obtained for etiocholanolone (Etio), androsterone (A), 16(5α)-androstenol (16EN) and 5β-pregnanediol (PD) in the urine specimens of the top-level soccer players residing in the six surveyed countries.

The carbon isotope ratios of androsterone and etiocholanolone are usually determined to prove doping with testosterone or a testosterone prohormone, because both compounds originate in the same androgen biosynthetic pathway (fig 1) and are excreted in the urine at relatively high concentrations compared to the other androgens. The sensitivity of IRMS may be the limiting factor in the accurate determination of low-level testosterone metabolites such as 5α-androstane-3α,17β-diol and 5β-androstane-3α,17β-diol. The metabolism of an exogenous anabolic steroid in humans will cause depletion in ^13^C of the steroid itself or of its metabolites in urine specimens, since the primary source of synthetic preparations are phytosterols extracted from ^13^C depleted plants (C3 plants), mainly soy and Mexican yam. During the drug washout period, the ^13^C/^12^C ratio of these compounds will progressively return to the natural background values characteristic of the diet of the individual. Ultimately, the variation in the ^13^C/^12^C ratio of the testosterone metabolites will depend on ^13^C depletion of the pharmaceutical preparation administered, as well as the athlete’s baseline values and individual metabolism.^12^ In our study, we focused on populations representative of top-level athletes for whom a very low prevalence of doping was assumed. Figure 2 shows the ^13^C/^12^C ratio expressed in δ^13^C values (‰) of androsterone and etiocholanolone together with 16(5α)-androstenol and 5β-pregnanediol. Of note, we did not find any evidence against the assumption that the δ^13^C values were normally distributed for each target steroid. Comparisons between the different groups show that Italian and Swiss populations have more negative δ^13^C values for all the tested steroids. At the same time, the Japanese soccer players exhibited intermediate δ^13^C values compared to those of the European countries and the group comprising ARG, SAF and UGA. For all the studied steroids, Argentinean players and players of African countries displayed a similar enrichment in ^13^C (p>0.2).

The δ^13^C values measured for the target compounds in each of the 171 urine specimens ranged from −17.2‰ to −25.2‰, thus reflecting the extent of isotopic fractionation as a function of the athletes’ diets and metabolisms. In order to understand these results, it is important to consider the period of time during which isotope ratios of steroids reflect one’s individual diet. Although in the case of human steroids, the turnover of stable carbon isotopes is still poorly understood, the mechanism of isotopic equilibration in steroids was nevertheless investigated in a recent preliminary study.^13^ Specifically, changes from standard European eating habits to a C4 plant-dominated diet revealed a common half life of about 17 days in the ^13^C enrichment process of the urinary steroids. In our study, we can assume that no significant shifts in the eating habits of the athletes occurred in the month prior to urine collection. Thus, it may be hypothesised that the excreted steroids were isotopically equilibrated with respect to the athlete’s diet. As stated previously, the distribution of carbon isotopes in animals reflects the relative abundance of C4 and C3 plants in their diet or those of the animals that are lower in the food chain. Compounds from C3 plants (eg, wheat, barley, oats, sugar beet, rye, cotton) generally have carbon isotopic values in the −35‰ to −22‰ range whereas compounds from C4 plants (eg, corn, millet, sugar cane, sorghum) exhibit δ^13^C values ranging from −8‰ to −20‰.^14^ In agreement with the data published recently,^15^ the mean δ^13^C values measured for androsterone were −19.0‰, −20.5‰ and −22.0‰ for SAF, JAP and ITA, respectively. This trend which is also confirmed for etiocholanolone, 5β-pregnanediol and 16(5α)-androstenol corresponds to an apparent higher consumption of C3 foodstuffs by European populations compared to other countries. Depletion in ^13^C in the steroids excreted by Italian and Swiss soccer players is reflected by more negative δ^13^C values according to the equation given previously.

It is important to note that ethnicity does not appear to contribute to variations in the ^13^C/^12^C ratio in steroids.^15^ ^16^ The natural variation in ^13^C/^12^C composition of steroids excreted in urine was recently described for men and women residing in Germany.^17^ For this European population, a metabolic ^13^C fractionation was observed between androsterone and etiocholanolone. It is believed that the isotopic fractionation between 5α and 5β metabolites originates from a kinetic isotope effect during the reduction step of testosterone.^18^ Our data show significant ^13^C depletion in etiocholanolone compared to androsterone for ARG, JAP, ITA and SWI populations (p<0.02). In contrast, both testosterone metabolites display comparable ^13^C enrichment for the athletes of SAF and UGA.

According to WADA guidelines, a Δδ^13^C difference of 3.0‰ or more between the δ^13^C values of testosterone metabolites and endogenous reference compounds (ERC) is consistent with the administration of exogenous steroids.^4^ This biomarker derived from the difference between isotope ratios constitutes a corrective factor for one’s individual diet. Endogenous steroids may be considered as ERC, if their carbon isotope values are not affected by the metabolism of xenobiotic compounds. Importantly, the technical document is applicable irrespective of the choice of the testosterone metabolite and the ERC chosen to calculate the Δδ^13^C value. In our study, it appears that the isotopic fractionation between androsterone and the ERCs is comparable for all individuals, regardless of their diet habit. Indeed, in the different groups of soccer players, no statistical difference was found between the Δδ^13^C values calculated from the difference between the δ^13^C values of androsterone and 16(5α)-androstenol or 5β-pregnanediol. In agreement with these findings, Piper *et al* observed no significant change in the difference between the ^13^C/^12^C ratios of 11β-hydroxyandrosterone, a product of the cortisol–cortisone metabolic pathway, and androsterone in urine samples of athletes residing in various geographical regions of the world.^19^

Table 1 lists the upper limit of the 99% confidence intervals of the differences in ^13^C/^12^C composition between testosterone metabolites and the ERCs for each athletes group. These intervals are determined by the sum of the mean value and threefold the standard deviation. Our data reveal discrepancies depending on the pair of steroids that is considered, thus illustrating the occurrence of isotope fractionation in steroid metabolism. Specifically, 99% confidence intervals for the differences in ^13^C/^12^C content between 5β-pregnanediol and androsterone are systematically lower than 2.5‰ whereas the 3‰ threshold value may not be suitable for steroid pairs based on the δ^13^C value of etiocholanolone and the ERCs.

**Table 1:** 99% Confidence intervals (CIs) of the Δδ^13^C values for each surveyed country calculated by the addition of the mean value and threefold SD

	99% CI			
	Δ(16EN-Etio)	Δ(16EN-A)	Δ(PD-Etio)	Δ(PD-A)
Uganda (n = 28)	2.8	2.3	3.0	2.3
South Africa (n = 30)	2.5	2.3	3.2	2.4
Switzerland (n = 31)	3.4	1.2	3.2	1.4
Italy (n = 19)	2.9	2.6	3.2	1.8
Argentina (n = 31)	2.6	1.9	2.7	1.7
Japan (n = 32)	3.2	2.9	2.5	2.3

All values are in δ^13^C_VPDB_ (‰).

16EN, 16(5α)-androstenol; A, androsterone; Etio, etiocholanolone; PD, 5β-pregnanediol.

### Conclusions

IRMS is already used in several antidoping laboratories to confirm the administration of testosterone. It is expected to become a universal analytical test in the forthcoming years. Today, the differences measured between the carbon isotope ratio of testosterone or its metabolites and an endogenous reference compound are considered as evidence of doping if one value is greater that 3‰, the threshold set by WADA. However, to validate the assay, the intervals of a reference population must also be determined.^20^ ^21^ This study assessed the sensitivity of the assay, which is limited by the imprecision in the measurements of the carbon isotope ratio and the natural isotope fractionation occurring during the metabolism of androgens. In agreement with previous investigations,^17^ ^20^ the intervals obtained from six different world populations of soccer players clearly show that the threshold of 3‰ is not suitable for all the Δδ^13^C values determined in our study. Indeed, such a threshold is too permissive for the Δδ^13^C value determined from 5β-pregnanediol and androsterone, thereby decreasing the diagnostic sensitivity of the test.

Our investigation suggests that different thresholds specific for each pair of steroids should be established. Of course, the evaluation of appropriate cut-offs should be assessed though interlaboratory collaborative trials. Such an approach would significantly increase the power of the test to show testosterone misuse. To further improve the strategy in the detection of testosterone administration, urine specimens for IRMS analysis may also be selected based on the abnormal variation in steroid time profile of the athlete.^22^ ^23^
